# Effects of pulse chirp on laser-driven proton acceleration

**DOI:** 10.1038/s41598-022-07019-4

**Published:** 2022-02-22

**Authors:** Alexander Permogorov, Giada Cantono, Diego Guenot, Anders Persson, Claes-Göran Wahlström

**Affiliations:** Department of Physics, Lund University, 22100 Lund, Sweden

**Keywords:** Laser-produced plasmas, Plasma-based accelerators

## Abstract

Optimisation and reproducibility of beams of protons accelerated from laser-solid interactions require accurate control of a wide set of variables, concerning both the laser pulse and the target. Among the former ones, the chirp and temporal shape of the pulse reaching the experimental area may vary because of spectral phase modulations acquired along the laser system and beam transport. Here, we present an experimental study where we investigate the influence of the laser pulse chirp on proton acceleration from ultrathin flat foils (10 and 100 nm thickness), while minimising any asymmetry in the pulse temporal shape. The results show a $\pm 10\%$ change in the maximum proton energy depending on the sign of the chirp. This effect is most noticeable from 10 nm-thick target foils, suggesting a chirp-dependent influence of relativistic transparency.

## Introduction

Laser-driven ion acceleration has been theoretically and experimentally studied for about two decades now. Due to their unique emittance and brightness, these sources have been suggested for various applications, from ultrafast radiography to hadron therapy and fast ignition in fusion applications, among others^1,2^. In the most practised acceleration scheme, known as Target Normal Sheath Acceleration (TNSA)^3^, an intense laser pulse ($I > 10^{18}$ W/cm$^2$) irradiates a solid target, and it formes a thin, overdense plasma (having an electron density $n_e$ greater than the critical density $n_c = \epsilon _0 m_e \omega ^2/e^2$, with $m_e$ and *e* the electron mass and charge, $\epsilon _0$ the vacuum permittivity and $\omega$ the laser frequency) through which it cannot propagate. Electrons on the irradiated side of the target gain energy in the laser field, cross the bulk and exit from the rear surface, forming a charged sheath. As a result, the associated strong electrostatic field ($\sim$ TV/m) ionises water and hydrocarbon contaminants present on the target surface and accelerates the ions along the target normal direction. Provided that the plasma gradient at the irradiated surface remains steep, an analogous sheath field is formed on the front side of the target, and protons are also accelerated in the backward normal direction^4^.

One of the main challenges towards the use of TNSA-driven proton beams in real applications is to improve the conversion from the laser energy to the number and energy of the sheath-forming electrons, and consequently to the maximum energy of the accelerated ions^5^. This generally involves altering the electron dynamics at the front surface of the target, by playing either with the target thickness^6^ and morphology^7^, or with the laser pulse properties (energy^8^, duration^4,9,10^, temporal profile^11–18^). Typically, the presence of a controlled amount of pre-plasma (with density rapidly decreasing below the critical value) at the target surface results in more efficient electron heating, as the laser pulse interacts with a larger volume of plasma electrons and resonant or stochastic absorption processes can take place^19,20^. Such a pre-plasma can be produced by irradiating the target with either a tailored pre-pulse^15–18^ or by the ns-long pedestal produced by Amplified Spontaneous Emission (ASE) in chiped-pulse-amplification laser systems^13,14^. In both cases, the energy deposited on target and the duration of the pre-plasma expansion determine the density profile for the main laser-plasma interaction. Optimal proton acceleration is achieved when the target is thick enough that the rear surface is not affected by the formation of pre-plasma on the front one^13^.

A modification of this approach is to alter the temporal profile of the main pulse itself. Recent numerical^21,22^ and experimental^23,24^ works have demonstrated that deviations from an ideal Fourier transform-limited pulse can lead to an increase of the cut-off energies of the laser-accelerated protons. However, there are still ambiguities about which pulse shape, and the related absorption process, optimises the proton acceleration. On the one hand, a laser pulse with fast rising edge penetrates through an ultrathin solid plasma, deeper than a pulse with a slow rising edge, because the higher instantaneous intensity leads to a lower plasma frequency ($\omega _p \propto \gamma ^{-1/2}$, with the Lorentz factor depending on the intensity of the laser field). In other words, the increase of the critical density in the relativistic regime ($\gamma n_c > n_c$, calculated when $\omega _p = \omega$), is affected by the instantaneous intensity, depending on the pulse shape. A deeper penetration hinders the heating at the front surface and has a negative effect on proton acceleration^21^. A similar result on electron heating has also been reported for negatively chirped pulses^25^. On the other hand, a laser pulse with a slow rising edge can gently ionise the target and produce a pre-plasma where absorption of the rest of the pulse is enhanced^23^. Yet it has been suggested that if a pre-plasma is already present, the ponderomotive force associated with the stronger gradients of a fast rising pulse would result in a better absorption and higher proton energies^22^. Finally, a recent experiment in the PW regime has reported a $40\%$ enhancement of the maximum proton energy for an unchirped, slow rising pulse, further increased to a twofold enhancement if combined with a negative chirp^24^.

In this article, we present our experimental results on the effects of laser pulse chirp on protons accelerated from nm-thick flat foils. We made sure that the pulse delivered to the target, at high intensity, was symmetric by carefully monitoring its spectral intensity and phase. In this way, we excluded any influence of the pulse shape and focused only on the role of the chirp. We found that the maximum proton energy depends on the sign of the chirp, indicating a more efficient absorption in the case of a positive chirp. The same trend for the proton energies is observed in both forward (FWD) and backward (BWD) target normal directions. However, the effect in the FWD direction is not detected when increasing the target thickness, suggesting that laser-induced relativistic transparency (RIT) may play a role in the acceleration of electrons through 10 nm-thick foils^21,26–30^.

## Results

Thin, free-standing carbon foils of 10 and 100 nm thicknesses were irradiated at $45^\circ$ incidence by a p-polarised, 350 mJ, 35 fs laser pulse, with a peak intensity at focus of $\sim 6.5 \times 10^{19}$ W/cm$^2$. Protons accelerated normally to the target surfaces were detected by a Thomson Parabola spectrometer in the FWD direction, and by a dipole magnet spectrometer in the BWD direction. The light transmitted during the interaction along the laser axis was measured with a Spectralon screen. A schematic illustration of the experimental setup is provided in “Methods”, Fig. 4.

**Figure 1:**
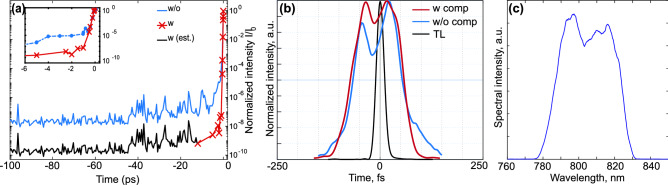
(**a**) Temporal contrast measured by a third-order cross-correlator, with and without DPM. The inset zooms in the last few ps before the main pulse, after the triggering of the DPM. The black curve is an estimation of the contrast when the signal is below the sensitivity of the cross-correlator. (**b**) Temporal shape retrieved by FROG on high-power shots, for the fully compressed pulse (transform-limited, TL), and for a chirped pulse ($b \approx +4$) with and without compensation of the asymmetry. (**c**) The spectrum of the laser pulse.

To ensure that the intense laser pulse interacts with a steep, overdense plasma, we employed a double plasma mirror (DPM) to reduce the intensity of the ASE pedestal below the ionisation threshold of the target. Figure 1a shows the contrast ratio measured by a third-order cross-correlator, which reaches $10^{-10}$ on the 100s-of-ps timescale and $10^{-8}$ at 1 ps before the main pulse. Although the intensity rapidly increases in the region of the coherent pedestal (later than $-1$ ps), the scale length of the produced pre-plasma is short enough to still allow for TNSA in the BWD direction^31^.

We used a single-shot frequency-resolved optical gating setup (FROG)^32^, arranged in the experimental chamber before the last focusing optic, to retrieve the temporal shape of the full-energy pulse right before the interaction with the target. This allowed us to characterise the pulse in terms of chirp and asymmetry (or skewness). The chirp parameter is calculated from the pulse duration $\tau$ and temporal phase $\varphi (t)$ as $b = \tau ^{2} \partial ^2\varphi (t)/\partial t^2$. When the pulse is negatively chirped ($b < 0$), its instantaneous frequency decreases with time, and vice versa in the case of positive chirp ($b > 0$). Chirp is introduced to the laser pulse when it is temporarily stretched, for example, by changing the separation between the gratings in the pulse compressor, which is the final part of a chirped-pulse-amplification-based laser system. By increasing or decreasing the distance with respect to the optimal separation, we achieve the same pulse duration but opposite sign of the chirp.

Figure 1b compares an optimally compressed pulse (black line) and a chirped one (blue line). One can note that besides the longer duration, the chirped pulse is asymmetric, with a dip in the middle and a flap on the falling edge. The dip is due to the shape of the laser spectrum, which is shown in Fig. 1c. The more the pulse is chirped, the more its temporal shape follows that of the spectrum, hence its symmetry needs to be monitored during the experiment, too. The additional asymmetry is a known consequence of imperfections of the optical and mechanical components as well as small alignment errors in the compressor, which means that moving the gratings gives rise to higher order terms in the pulse phase^33^. The most relevant term in the expansion of the spectral phase is the third order dispersion (TOD, $\propto \partial ^3 \phi /\partial \omega ^3$), which we compensated for with an acouto-optic dispersive filter (Dazzler)^34^. The final pulse asymmetry can be quantified with the skewness parameter^35^ $S= m_3/m_2^{3/2}$, where $m_k=\int _{t_a}^{t_b} (t-t_0)^k I(t) dt / \int _{t_a}^{t_b} I(t) dt$ are the *k*-th momenta of the pulse, calculated around its centroid $t_0$. The pulse has a steep rising (or falling) edge when *S* is positive (or negative, respectively), while $S=0$ corresponds to a symmetric pulse. When we varied the chirp and neglected the possible changes in the spectral shape, *S* ranged between $-1.0$ and 1.8. After correcting the pulse asymmetry, instead, the skewness was reduced to $\pm 0.3$. For the asymmetric pulse in Fig. 1b, $S=-0.8$. The red curve, instead, represents the symmetric shape achieved after compensation of the TOD, having $S=0.1$.

**Figure 2:**
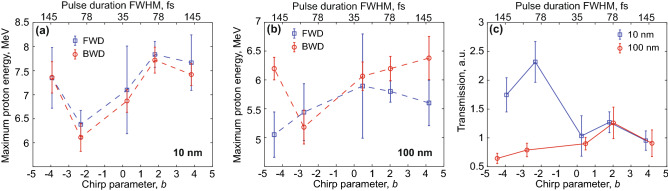
(**a**,**b**) Proton cut-off energies in forward (FWD) and backward (BWD) directions obtained from 10 nm- (**a**) and 100 nm-thick (**b**) carbon foils. (**c**) Transmitted laser light measured by the Spectralon, for both target thicknesses. In all panels, error bars represent the mean standard error calculated on the average points.

Figure 2a,b shows the dependence of the maximum proton energy, measured in FWD and BWD directions, on the laser chirp, for both thicknesses of the foil. Comparable energies in both directions support that the temporal contrast is high enough even for the 10 nm-thick foil to remain essentially unperturbed until the arrival of the main laser pulse (panel (a)). If a pre-plasma is formed at the front surface, its extent is negligible since the acceleration mechanism is similar on both sides of the target^4,31^. The energies obtained from the 10 nm foil are higher than those from the 100 nm one (panel (b)), compatible with the reduced thickness that favours electron recirculation and heating in the laser field^36^.

More importantly, from the 10 nm-thick foil we observe a clear effect of the sign of the chirp. On the one hand, the maximum proton energies increase when introducing a positive chirp, with a peak in the FWD direction at $7.8 \pm 0.3$ MeV, which is $10\%$ more than the average energy measured with a fully-compressed, unchirped pulse ($b=0$, $\tau \simeq 35$ fs). On the other hand, the FWD cut-off energy decreases by $10\%$ when a moderate negative chirp is used ($b=-2.3$, $\tau \simeq 80$ fs), and it raises again when the chirp is further increased. Remarkably, for the thinnest target the reduction of the laser intensity, due to the increased pulse duration, does not appear to impair the proton acceleration within the range of our scan. While for the 10 nm foil the energies in the BWD direction closely follow those in the FWD direction, we observe a different trend with the 100 nm foil. In this case, the energies show a similar dip for negative chirp, and a small enhancement for positive chirp, only in the BWD direction (corresponding to a $-14\%$ and $+2\%$ variation compared to the energy obtained with the unchirped pulse). In the FWD direction, instead, the maximum energies decrease for longer pulse duration, regardless of the sign of the chirp, and the maximum energy is achieved with the shortest (unchirped) pulse, in correspondence with the highest intensity on target. This result is consistent with previously reported experimental works employing a similar target thickness^4^.

A dependence on both the target thickness and the chirp is also noticeable in the amount of laser light transmitted during the interaction, and presented in Fig. 2c. Here, for the thinnest foil we observe a peak in transmission for negative chirp, in correspondence of the same chirp where the proton energy drops. Inversely, the 100 nm foil shows very little variation of the transmitted light for all the investigated values of chirp.

## Discussion

Our experimental results show that the maximum energy of laser-driven proton beams is affected by the sign of the chirp. For extremely thin targets (10 nm), a dependence is equally visible in the FWD and BWD directions, and it is found to correlate with the amount of light transmitted through the target. However, the influence of the chirp on the maximum energy in the FWD direction and on the laser transmission vanishes when the target thickness is increased to 100 nm. In this section, we examine possible mechanisms that might be related to these experimental observations. First, we discuss how the thickness of the foil could explain the different trends of the proton energy observed in the FWD and BWD direction with the 100 nm target. Then, we discuss how the chirp, and particularly its sign, can affect electron heating by changing the transmission of the pulse through the overdense plasma, and the field that is formed at the front surface of the target.

We already mentioned that similar trends of the proton energies in the BWD and FWD direction suggest a similar acceleration mechanism on both sides of the target. From the results with the 10 nm-thick foil, it appears that a more efficient heating (hence absorption of the laser energy) occurs on the front side when chirp is applied (except for $b \approx -2$). The fact that the chirp-dependence is not observed in the FWD direction with the 100 nm-thick foil indicates that the thickness affects the generation of the accelerating sheath at the rear surface. This is more evident when we applied the largest amounts of chirp ($b \approx \pm 4.5$), and we obtained higher energies in the BWD direction compared to those in the FWD direction. There, the combination of electron propagation through the thicker target and of the reduced laser intensity determines the broad, symmetric shape of the blue curve in Fig. 2b. On the other side, instead, electrons moving away from the front-surface plasma retain the dependence on the chirp, accelerating the protons with a trend similar to the one observed with the 10 nm-thick foil.

With regard to the details about how the sign of the chirp affects absorption and proton acceleration, a first important consideration is that the effect of the chirp on the proton energies, although not negligible, is smaller than what is obtained when varying the pulse shape. Previous experiments, in fact, reported large energy enhancements when a pulse with a slow leading edge was used instead of a symmetric one ($+70\%$ in Tayyab et al.^23^, and $+40\%$ in Ziegler et al.^24^). In the first case, the skewed shape was the result of not compensating the high order terms of the spectral phase when moving the gratings in the compressor. While this caused the effects due to chirp and asymmetry to be entangled, Ziegler et al.^24^ demonstrated that changing the shape was more effective than changing only the chirp (the proton energy increased by $40\%$ in the first case, versus $20\%$ in the second). For these reasons, a residual asymmetry of the chirped pulses could dominate over the effect of the chirp. However, the measurements of the pulse shape allow us to rule out this scenario, because the skewness calculated for the chirped pulses that produce the largest variations of the proton energy, is comparable with the skewness of the unchirped pulse. For $b \approx \pm 2$, $S=-0.07$ and $S=-0.33$ for the 10 nm-thick foil, while $S=0.34$ and $S=0.13$ for the 100 nm-thick foil. For the unchirped pulse ($b=0$), instead, $S=0.3$ in both cases. These values also demonstrate that the sign of the residual asymmetry is not correlated with the trend of the maximum proton energy. For example in Fig. 2a, both the largest and smallest values of proton energy are observed with $S<0$.

Having excluded effects due to the asymmetry, we now discuss the dependence on chirp of the laser transmission. It is known that the optical response of a plasma irradiated by a relativistically intense laser pulse can be described, in a first approximation, by correcting the electron mass with the Lorentz factor $\gamma =(1+a_0^2/2)^{1/2}$, where $a_0$ is the normalised amplitude of the laser field. As already mentioned in the introduction, this causes a decrease of the plasma frequency, with the result that the laser can propagate inside the plasma in the region where $\omega > \omega _p/\gamma ^{1/2}$, corresponding to an increased critical density $\gamma n_c > n_c$. In this effect, known as relativistic induced transparency (RIT), the penetration of the laser is counteracted by the local increase of the electron density at the surface of the plasma, piled up by the ponderomotive force. Therefore, RIT is usually observed with ultrathin targets and high laser intensities (small electron density and large $\gamma$). For an ultrathin plasma of thickness *l* and electron density $n_e$, the onset of RIT has been estimated when $a_0 \gtrsim \pi n_e l/(n_c \lambda )$, $\lambda$ being the laser wavelength^37^. This condition indicates that carbon foils below 10 nm of thickness become transparent at laser intensities just below $5\times 10^{20}$ W/cm$^2$, but additional processes contributing to plasma heating and expansion have been demonstrated to relax this threshold^38,39^. In the experimental context, the target thickness corresponding to the onset of RIT will depend on the angle of incidence, laser polarisation, temporal contrast, etc., giving rise to a space of variables were multiple (hybrid) acceleration mechanisms emerge^26–30^. Recently, Poole et al.^26^ have reported evidence of RIT on 40 nm-thick foils irradiated at $10^{21}$ W/cm$^2$, which is a  16-time higher intensity than the one used in our experiment. Since $a_0 \propto I^{1/2}$, their results reinforce that the 10 nm-thick foil can become transparent at our intensities.

While the occurrence of RIT primarily depends on the target thickness, the presence of chirp-dependent effects may explain the dip of the proton energy observed at negative chirp, in conjunction with the peak in transmission measured with the Spectralon. If the foil becomes transparent too early, electrons are not efficiently heated by the most intense part of the laser pulse, and proton acceleration becomes less efficient. A similar process explains why targets that are too thin undergo RIT and produce low energy protons^27,39^. Numerical simulations have indicated that the piling up of electrons at the front surface of the target can be affected by chirp, with a higher accumulation (hence less transmission and more efficient heating) for a positive chirp^21^. Similarly, a higher transmission of negatively-chirped pulses has been reported in other numerical works^25,40^, even though in some of these studies the changes of the chirp are not sustained by a consistent change of the pulse duration (or of the spectral width). Note that by simply estimating the laser penetration with the formula for the skin depth, $l_s=c(\omega _p^2/\gamma -\omega ^2(t))^{-1/2}$, and including the chirp in the expression for the laser frequency, the difference in between the points at $b=\pm 2$ is at most $~2\%$. This is because such calculation neglects the self-consistent evolution of the plasma density during the arrival of the laser pulse, which is even more complex if the target already includes some underdense pre-plasma at the front surface^25^.

It is also worth mentioning that if the target thickness is such that RIT occurs at the peak of the laser pulse, strong volumetric heating and ion acceleration can take place. In this process, known as break-out afterburner (BOA)^41^, ions are accelerated along the laser axis, similarly to what happens with radiation pressure acceleration (RPA)^42^. The transition from TNSA to these other acceleration mechanisms has been observed in recent experiments with ultrathin targets and laser intensities exceeding $10^{20}$ W/cm$^2$^27,28,30^. Below these intensities, RPA is not efficient in case of linear polarisation, and TNSA remains the dominant acceleration mechanism^43^. In our experiment we do not expect RPA or BOA to contribute to proton acceleration: Not only the laser-target interaction conditions were not optimised for these mechanisms, but also our diagnostics are set along the target normal, preventing us from measuring the ion spectra along the laser axis.

Another effect that may occur in addition to the ones above, is that the chirp modifies the standing wave (SW) that is formed normally to the front surface of the target by the interference of the incident and reflected laser pulses^44^. The resulting electric field is at the base of the “capacitor model” firstly introduced to explain electron heating at the steep surface of an overdense plasma^45^. Electrons are pulled out in vacuum by the oscillating capacitor field and then re-enter the plasma after half a cycle, delivering their energy^2^. In recent works, chirped laser pulses have been studied in relation to acceleration mechanisms other than TNSA, which may offer higher conversion efficiencies from laser to ion energies in the newly-available PW regime^46,47^. When the incident laser field is chirped, the nodes of the SW move in time, with the direction determined by the sign of the chirp: toward the target if the chirp is positive, and away from it in the opposite case. It is worth noting that in the case of p-polarisation, bunches of electrons cannot be locked in the nodes of the SW and steadily accelerated during the motion of the nodes, as done for example in Magnusson et al.^46^. Whether the electrons gain energy in the SW and cross the target again will depend on the initial phase with which they entered the accelerating fields from the plasma^48^. But we believe that if the nodes move towards the target, the position where the electric field changes its sign remains closer to the plasma than if the nodes move away. This can lead to a more efficient injection of electrons in the overdense plasma, which corresponds to a higher absorption. The consequent increase of the proton energy is visible in both FWD and BWD only if the target foil is thin, allowing for electron recirculation, and if it does not become transparent during the interaction. This may also explain why, if the nodes are moving in a unique direction, the same signature of the chirp is observed along both directions of the target normal axis.

**Figure 3:**
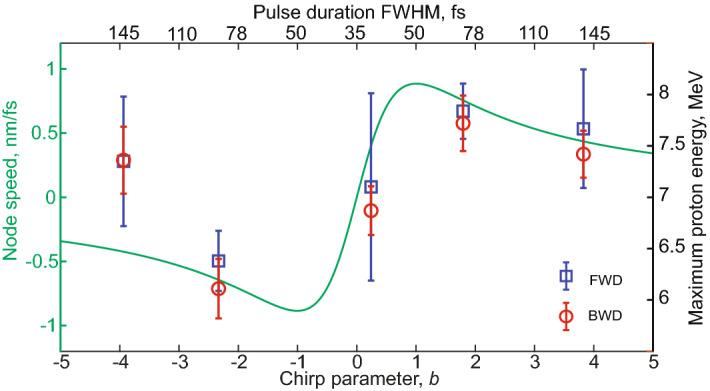
Velocity of the nodes of the SW, as a function of the chirp in the same range of the experiment (green curve), together with the proton energies detected from the 10 nm-thick foil.

We have calculated the average velocity of the nodes of the SW, for chirp values in the same range as used in the experiment. For a Gaussian pulse, this is expressed by the first derivative of the instantaneous wavelength, $\partial \lambda /\partial t= B \lambda _0/(1+Bt)^2$, with $\lambda _0$ the central wavelength of the pulse and $B=\lambda _0 b/(\pi c \tau ^2)$, then averaged over the duration of the laser pulse. As shown in Fig. 3, the variation of the node velocity follows the same trend as the proton energies observed with the 10 nm-thick foil. Clearly, the absolute value of the node velocity cannot correspond to the proton velocity, as their acceleration is first mediated by electrons. Nevertheless, the existence of a similar trend is worth exploring with the assistance of numerical simulations or with dedicated experimental works.

## Conclusion

In this study, we focused on the effect of the laser chirp on proton acceleration, by removing higher order terms in the pulse spectral phase originated in the pulse compressor. We found a clear influence of the sign of the chirp on the maximum proton energy and on the transmitted light from the 10 nm-thick foil, while the chirp-dependence was detected only in the BWD-accelerated protons when using the 100 nm-thick foil. This indicates that the role of the chirp on energy absorption or electron heating is very subtle, its study requiring ultrathin targets, high temporal contrast and accurate control of the pulse shape over a sub-fs time scale and at high intensity. In this regime, relativistic transparency can take place and give rise to hybrid acceleration mechanisms where unexpected trends in the proton beam energy, profile or direction can arise^26,27^. We have also discussed about the effect of the chirp on the standing wave formed in front of the target. The nodes acquire a velocity that depends on the sign of the chirp with the same trend as the measured proton energies. Despite not providing an explanation for the evolution of target absorption or of the accelerating fields, these observations motivate future numerical and experimental works. In particular, we recommend investigating whether changing the laser polarisation and the angle of incidence would reduce the weight of heating mechanisms that are usual for TNSA, and in turn favour the emerging of other processes. Diagnostics for probing the relativistic transparency, or the emission of proton beams away from the target normal directions, would allow monitoring any transition from TNSA to alternative acceleration mechanisms, such as RPA^27,30^ or BOA^28^. Finally, the support from particle-in-cell simulation, able to resolve the details of the electron dynamics during the interaction with the chirped laser pulse, would push the understanding of the underlying physics to a new level.

## Methods

### Experimental setup

The TW laser at Lund Laser Centre is a chirped-pulse-amplification system, capable of delivering up to 2 J of energy per laser pulse before compression. The spectrum is centred at a wavelength of 800 nm, with a FWHM of 40 nm (Fig. 1c). The shortest pulse duration, achieved with a grating-based compressor, is 35 fs. The transport line includes a deformable mirror to remove wavefront aberrations and to improve the focusability of the beam. In the present experiment, the p-polarised laser pulse reaching the target had an energy of 350 mJ and it was focused at $45^\circ$ angle of incidence with a spot size of $\sim 3$ μm FHWM. The resulting peak intensity was $6.5 \times 10^{19}$ W/cm$^2$, corresponding to a normalised field amplitude $a_0 \simeq 0.85 \lambda _{\upmu \text {m}}(10^{-18}I_{\text {W/cm}^2})^{1/2} = 5.5$.

We used a double plasma mirror (DPM) to ensure a high temporal contrast of the laser pulse^49^. The DPM consists of a pair of confocally arranged off-axis parabolic mirrors, with two anti-reflection-coated glass plates placed before and after the focus. The intensity pedestal due to amplified spontaneous emission (ASE) is transmitted through the glass surface, while depositing energy in it. When the amount of deposited energy has ionised enough electrons so that their density becomes overcritical for the laser wavelength, the remaining pulse is fully reflected. The temporal contrast as measured by a third-order cross-correlator is shown in Fig. 1a. It shows that when using the DPM, the intensity of the radiation at $-10$ ps before the main pulse is reduced by two orders of magnitude. The contrast ratio is better than $10^{-8}$ until $-1$ ps before the peak.

**Figure 4:**
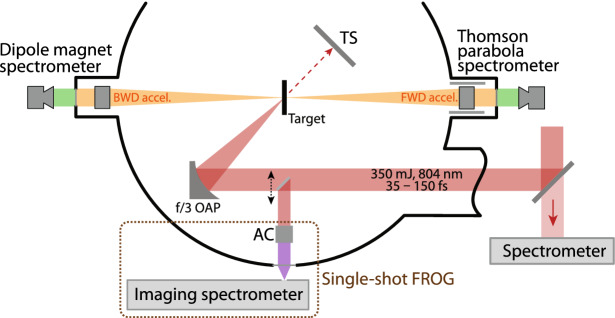
Illustration of the experimental setup. TS denotes the Spectralon screen detecting transmitted laser light, OAP is the focusing off-axis parabola, and AC stands for auto-correlator.

The targets consisted of amorphous carbon foils (ACF-Metals) of 10 and 100 nm thickness, having a mass density of 2.0 g/cm$^3$. Protons accelerated along the normal axis of the target were detected in the FWD direction by a calibrated Thomson Parabola spectrometer, having a micro-channel plate with a phosphor screen as a detector. Protons accelerated in the BWD direction, instead, were dispersed by a dipole magnet and detected by a scintillator screen. A 12.5 μm-thick Aluminium foil was placed in front of the scintillator, for light shielding and stopping of heavier ions. In addition, we put a scattering Spectralon screen (Labsphere) behind the target, along the laser axis, to measure the intensity of the transmitted laser light. The signal diffused by the screen was detected by a CCD camera equipped with 800 nm-bandpass filter and calibrated neutral density filters. The setup of the experimental chamber is given in Fig. 4.

### Pulse shape control

Before investigating the effect of the chirp on the accelerated protons, we characterised the shape of the pulse reaching the target with dedicated, high-power shots. To do so, the laser pulse was intercepted before the focusing parabola in the experimental chamber, attenuated by a factor of 35 by reflection from ZnSe and BK-7 wedges and sent to a single-shot second-harmonic autocorrelator (AC in Fig. 4). Here, the incoming beam was split in two paths with a knife-edge reflective prism, and recombined later on a 300 μm-thick KDP crystal. The non-collinear harmonic signal was then relayed outside the experimental chamber and focused on an imaging spectrometer. The auto-correlator and the spectrometer combined together formed a single-shot FROG^32^. Retrieval of the FROG traces allowed us to reconstruct the temporal and spectral complex envelopes of the laser pulse as it was incident on the target, and to have insight on the necessary adjustments required to make the shape symmetric.

We also used a calibrated, near-infrared spectrometer to measure the laser light leaking from a broad-band dielectric mirror located at the entrance of the experimental chamber. In this way, we could double-check the spectral intensity of the pulse as reconstructed from the FROG trace, and monitor the pulse spectrum in those shots where the FROG was by-passed.

During the experiment we varied the chirp of the pulse, while keeping the temporal shape as symmetric as possible. A chirped laser pulse is temporarily stretched, so we introduced chirp by changing the separation of the gratings in the pulse compressor. With respect to the optimal separation that produces the shortest pulse, increasing the distance between the gratings produces a negatively chirped pulse ($b<0$ and instantaneous frequency decreasing with time). Decreasing the distance produces the opposite effect. The pulse duration increases in both cases, since it is independent from the sign of the chirp ($\tau \propto \sqrt{1+b^2}$ for a Gaussian pulse). Using the pulse temporal phase $\varphi (t)$ obtained from the FROG measurements, we calculated that in the experiment the chirp parameter *b* varied between $\pm 4.5$, corresponding a maximum pulse duration of $\sim 150$ fs. Referring to the values of pulse duration indicated in the top-axis of Fig. 2, the peak intensity decreased to $2.9 \times 10^{19}$ W/cm$^2$ for 78 fs and to $1.6 \times 10^{19}$ W/cm$^2$ for 145 fs, respectively.

The asymmetry, instead, is related to the shape of the laser spectrum and to the third order term of the Taylor expansion of the spectral phase (third order dispersion, TOD). To minimise the pulse asymmetry, we minimised the TOD and kept a symmetric spectrum with a Dazzler (Fastlite)^34^, a digital programmable acousto-optic modulator that can introduce an arbitrary amount of spectral phase in the Taylor expansion, as well as change the intensity of different parts of the spectrum. Since the Dazzler is placed early in the laser system, before amplification and compression, we first measured the spectral phase and intensity of the pulse with the FROG, and then applied the necessary changes to minimise the asymmetry. The amount of TOD applied with the Dazzler ranged between $\pm 15$k fs$^3$, depending on the grating separation. The residual TOD measured by the FROG after shape compensation always lied between $\pm 2$k fs$^3$. We finally calculated the skewness parameter *S* from the retrieved temporal shape.

Note that the chirp can be changed with the Dazzler too, as it is related to the second term of the Taylor expansion of the spectral phase (group velocity dispersion, GVD). However, we restrained from doing so in our experiment because it entailed the risk of damaging the amplifiers later in the laser chain.
